# Cadmium resistance and uptake by bacterium, *Salmonella enterica* 43C, isolated from industrial effluent

**DOI:** 10.1186/s13568-016-0225-9

**Published:** 2016-08-04

**Authors:** Zaman Khan, Abdul Rehman, Syed Z. Hussain, Muhammad A. Nisar, Soumble Zulfiqar, Abdul R. Shakoori

**Affiliations:** 1 Department of Microbiology and Molecular Genetics, University of the Punjab, New Campus, Lahore, 54590 Pakistan; 2 Department of Chemistry, SBA School of Science and Engineering (SBASSE), Lahore University of Management Sciences (LUMS), DHA, Lahore Cantt, 54792 Pakistan; 3 Department of Microbiology, Govt. College University Faislabad, Faislabad, Pakistan; 4 School of Biological Sciences, University of the Punjab, New Campus, Lahore, 54590 Pakistan

**Keywords:** Cd-resistance, *S. enterica* 43C, Biosorption, Glutathione, Metallothionein, *smtAB* gene

## Abstract

Cadmium resistant bacterium, isolated from industrial wastewater, was characterized as *Salmonella enterica* 43C on the basis of biochemical and 16S rRNA ribotyping. It is first ever reported *S. enterica* 43C bared extreme resistance against heavy metal consortia in order of Pb^2+^>Cd^2+^>As^3+^>Zn^2+^>Cr^6+^>Cu^2+^>Hg^2+^. Cd^2+^ stress altered growth pattern of the bacterium in time dependent manner. It could remove nearly 57 % Cd^2+^ from the medium over a period of 8 days. Kinetic and thermodynamic studies based on various adsorption isotherm models (Langmuir and Freundlich) depicted the Cd^2+^ biosorption as spontaneous, feasible and endothermic in nature. Interestingly, the bacterium followed pseudo first order kinetics, making it a good biosorbent for heavy metal ions. The *S. enterica* 43C Cd^2+^ processivity was significantly influenced by temperature, pH, initial Cd^2+^ concentration, biomass dosage and co-metal ions. FTIR analysis of the bacterium revealed the active participation of amide and carbonyl moieties in Cd^2+^ adsorption confirmed by EDX analysis. Electron micrographs beckoned further surface adsorption and increased bacterial size due to intracellular Cd^2+^ accumulation. An overwhelming increase in glutathione and other non-protein thiols levels played a significant role in thriving oxidative stress generated by metal cations. Presence of metallothionein clearly depicted the role of such proteins in bacterial metal resistance mechanism. The present study results clearly declare *S. enterica* 43C a suitable candidate for green chemistry to bioremediate environmental Cd^2+^.

**Electronic supplementary material:**

The online version of this article (doi:10.1186/s13568-016-0225-9) contains supplementary material, which is available to authorized users.

## Introduction

Heavy metal pollution has become a major environmental problem worldwide (Ali et al. 2013). Some heavy metals including arsenic (As), cadmium (Cd), mercury (Hg) and chromium (Cr) are called non-essential metals as these are not needed for the survival of organisms and are toxic in low concentrations. Cd is highly toxic, non-essential and non-biodegradable heavy metal with half-life of 20 years (Martelli et al. 2006; Aksoy et al. 2014; Xu et al. 2014; Vinodini et al. 2015). It is soft, silver-white, electropositive metal having atomic number 48, atomic mass 112.41, density 8.64 g/cm^3^ and melting point 321 °C. It is divalent in all of its stable compounds and found abundantly in nature in the form of CdS. It may also combine with ammonia and cyanide to form Cd(NH_3_)_6_^−4^ and Cd(CN)_4_^−2^, respectively. Various industrial processes such as mining, electroplating, stabilizing plastics, manufacturing batteries, alloy, pigment, cement, fossil fuel combustion, municipal and sewage sludge incineration and high phosphate fertilizers are responsible for the release of huge amount of Cd^2+^ in the environment (Klaassen et al. 2009; Yazdankhah et al. 2010; Liu et al. 2013). According to an estimation 30,000 tons Cd^2+^ is released in our environment annually (Nriagu and Pacyna 1988).

Cd and almost all of its compounds are water soluble and hence easily gain entry in human food chain. No physiological role of cadmium in human cellular metabolism has been reported so far and it is extremely toxic in very minute quantity (Aksoy et al. 2014; Xu et al. 2014). It has also been reported to cause osteoporosis and fractures, anemia, eosinophilia, anosmia, apoptosis, diabetes mellitus, oncogenes activation, Itai–Itai disease and chronic pulmonary problems (Waisberg et al. 2003; Edwards and Prozialeck 2009; Yazdankhah et al. 2010).

No treatment for cadmium toxicity has been approved so far (Zhai et al. 2015). Several chemical and physical methods are used to remove cadmium from industrial effluent prior to release the effluent into the environment but all these methods are expensive and less effective (Kurniawan et al. 2006). So the use of living and dead microbial biomass is gaining attraction to remove heavy metals from environment due to less expensive, effective, time efficient and environmental friendly strategy (Feng and Aldrich 2004; He et al. 2011; Huang et al. 2014). Bacteria remove heavy metal ions including Cd^2+^ from the environment either by metabolism independent adsorption on their cell walls or metabolism dependent intracellular accumulation (Gadd 1990; Srinath et al. 2002; Vargas-García et al. 2012). Hyperaccumulation of Cd^2+^ has been reported to disturb the cell physiology by reactive oxygen species (ROS) production and disruption of bacterial respiratory proteins (Gibbons et al. 2011; Zeng et al. 2012). Bacteria have evolved several resistance mechanisms including efflux transport, precipitation, transformation and intracellular sequestration by metallothionein, glutathione and other thiol containing compounds to combat the negative effects of heavy metal ions intracellular accumulation (Nies 2003; Intorne et al. 2012; Maynaud et al. 2014).

The present study is aimed at the isolation and characterization of Cd^2+^ resistant bacterium from industrial wastewater and its potential use to remove Cd^2+^ from aqueous environment. The effect of culture conditions with reference to pH, temperature, initial Cd^2+^ concentration, biomass dosage and co-metal ions on the Cd^2+^ processing capability of the bacterium was also measured. Adsorption data was evaluated by applying adsorption isotherm models, thermodynamic and kinetic studies. Adsorption and intracellular accumulation of Cd^2+^ were confirmed by FTIR, SEM and EDX analysis. The effect of Cd^2+^ stress on bacterial growth and cell physiology (change in glutathione and non-protein thiol levels) was also determined.

## Materials and methods

### Sample collection

Industrial wastewater samples were collected in sterilized screw capped bottles from different industrial areas of Sheikhupura and Kotlakhpat, Lahore, Pakistan. Some physiochemical parameters of wastewater samples viz., temperature (°C), pH and cadmium ion concentration (mM) were measured.

### Isolation and purification of cadmium resistant bacteria

To isolate cadmium resistant bacteria, 100 µL of wastewater sample was spread on Luria-Bertani (LB) agar (1 % NaCl, 1 % tryptone, 0.5 % yeast extract and 1.5 % agar) plates supplemented with 1 mM Cd^2+^. Bacterial growth was observed after incubation at 37 °C for 24 h. Single colony was picked with sterilized wire loop and re-streaked on Cd^2+^ supplemented LB agar plates and again incubated at 37 °C for 24 h. The process was repeated until the purified single colony obtained.

### Determination of minimum inhibitory concentration

The bacterial isolate was grown in minimal salt (MS) broth (1 % (NH_4_)_2_SO_4_, 0.086 % CaCl_2_, 0.15 % K_2_HPO_4_, 0.1 % KH_2_PO_4_, 0.1 % MgSO_4_, 0.02 % FeSO_4_ and 1 % glucose) supplemented with 1 mM Cd^2+^ at 37 °C for 24 h. Growth was observed by taking absorbance at 600 nm. The process was repeated with high concentrations of Cd^2+^ until the growth of the isolate was inhibited. The minimum Cd^2+^ concentration at which bacterial isolate did not show growth was considered as its MIC.

### Determination of optimum growth conditions

Optimum growth conditions of bacterial isolate were determined with respect to temperature and pH. To determine optimum temperature, isolate was grown in LB broth at different incubating temperatures viz., 20, 25, 30, 37 and 42 °C. After 24 h incubation, their absorbance was measured at 600 nm using spectrophotometer. To determine optimum pH, 250 mL flasks having 100 mL LB broth were prepared in six sets, each set containing three flasks, their pH were adjusted at 5, 6, 7, 8, 9 and 10 and autoclaved. These flasks were inoculated with 1 % freshly prepared culture of the isolate. After 24 h incubation at optimum temperature, absorbance was taken at 600 nm.

### Effect of cadmium on bacterial growth

Effect of Cd^2+^ on the growth of bacterial strain was determined by growing in the presence (1 mM Cd^2+^) as well in the absence (control) of Cd^2+^. MS broth (100 mL) was taken in a set of three 250 mL flasks, autoclaved, supplemented with 1 mM Cd^2+^, inoculated with 1 % of freshly prepared inoculum and incubated at 37 °C in shaking incubator at 100 rpm. In one flask no Cd^2+^ was added which worked as a positive control. Growth in each culture was determined every 4 h by taking absorbance of an aliquot (1 mL) at 600 nm. Growth curves were plotted between time and absorbance.

### Resistance to heavy metal ions

Resistance of bacterial isolate against heavy metal ions (zinc, lead, copper, chromium, arsenic and mercury) was determined by growing it in MS broth supplemented with respective metal ions. Stock solutions of 1 M concentration of heavy metal ions salts (zinc sulfate, lead nitrate, copper sulfate, potassium dichromate, sodium arsenate and mercuric chloride) were used. The concentration of metal ions was increased, 1 mM every time, in a stepwise manner until the growth of the isolate was inhibited. The bacterial growth was determined by taking optical density (OD) at 600 nm after 24 h of incubation at 37 °C.

### Physical, biochemical and molecular characterization

Bacterial isolate was identified on the basis of colony morphology and different biochemical tests such as Gram staining, catalase test, oxidase test, citrate utilization, fermentation of carbohydrates, H_2_S production, nitrate reduction, indole and urease test (Benson 1994). For molecular characterization, 16S rRNA gene was amplified through polymerase chain reaction (PCR) by using general primers (RS-1; 5′-AAACTCAAATGAATTGACGG-3′, RS-3; 5′-ACGGGCGGTGTGTAC-3′) (Rehman et al. 2007). Amplification of 16S rRNA gene was carried out by 35 cycles of denaturation at 94 °C for 1 min, annealing at 55 °C for 1 min and extension at 72 °C for 2 min followed by final extension at 72 °C for 5 min. The PCR product (approximately 0.5 kb) was purified through Fermentas GeneJet Gel Extraction kit (#K0691) and sequenced from Macrogen, Korea. The sequence obtained was compared with known sequences in NCBI database using NCBI BLAST tool (http://www.ncbi.nlm.nih.gov/BLAST) to identify the bacterial isolate. The accession number was obtained by submitting the sequence in the GenBank.

### Cadmium processing ability of bacterial isolate

To ascertain cadmium processing ability, the bacterial isolate was grown in 100 mL LB broth in 250 mL flask on shaking (100 rpm) at optimum temperature and pH. After 24 h of incubation the medium was supplemented with 1 mM Cd^2+^. In one flask Cd^2+^ was added in the same concentration (1 mM) but not inoculated with the organism, which worked as a control. The experiment was carried out in triplicate. Then 5 mL samples were taken out from each flask after 2, 4, 6 and 8 days. Bacterial cells were harvested through centrifugation at 3000 rpm for 5 min. The pellets [acid digested, 0.2 N HNO_3_ (1:1)] and supernatants were used for the estimation of Cd^2+^ by atomic absorption spectrophotometer at a wavelength of 228.8 nm. Each pellet was weighed and divided into two portions, one portion was washed thrice with 0.5 M EDTA to collect Cd^2+^ adsorbed on the cell surface and second portion was acid digested to release absorbed Cd^2+^. Total uptake values were calculated by subtracting the metal concentration in treated flasks from the metal concentration in control flask. Standard curve was prepared to calculate amount of metal ions in pellets and supernatants. Percentage increase and decrease in the amount of metal ions were also calculated.

### Removal of Cd^2+^ from industrial effluent

A lab experiment was set up to determine the efficacy of bacterial isolate to remove Cd^2+^ from industrial wastewater. Our set up (experiment design) consisted of three plastic containers; one container carrying 10 L distilled water along with 1.5 L bacterial culture grown to log phase, second container carrying 10 L industrial effluent along with 1.5 L bacterial culture while third container was carrying only 10 L industrial effluent. Experiment was carried out at room temperature (25 ± 2 °C) and 1 mM cadmium stress was maintained in each container. Samples (10 mL) were taken from each container after 2, 4, 6 and 8 days of incubation and cells were harvested by centrifugation at 3000 rpm for 5 min. Cell pellets were weighed and separated whereas supernatants were used to estimate the amount of Cd^2+^ in the wastewater and further removed by bacterial isolate.

### Cd^2+^ removal by heat-inactivated bacterial cells

Biosorption of Cd^2+^ by heat inactivated bacterial cells was studied by growing the isolate in 250 mL Enlermayer flask containing 100 mL LB broth medium at 37 °C in shaking incubator (150 rpm). After 24 h of incubation bacterial cells were inactivated by autoclaving at 121 °C and 15 LB pressure for 15 min. Dead bacterial cells were harvested by centrifugation at 10,000 rpm for 10 min. Cells were washed with autoclaved deionized distilled water thrice and dried at 80 °C in oven. Dead bacterial biomass (0.5 g) was added in flask containing 100 mL of 1 mM Cd^2+^ solution and incubated at 37 °C on shaking (150 rpm). After regular time intervals samples were collected and centrifuged at 3000 rpm for 5 min. Pellets and supernatants were used to estimate the quantity of Cd^2+^ by atomic absorption spectrophotometer. The experiment was performed in triplicate.

### Effect of temperature, pH, initial Cd^2+^ concentration, biomass dosage and co-metal ions on Cd^2+^ processing ability of bacterial biomass

To ascertain the effect of temperature and pH on the Cd^2+^ processing ability of the bacterial isolate, it was grown in LB broth to log phase in flasks on optimum conditions, then 1 mM Cd^2+^ stress was given in each flask and incubated at different incubating temperatures (25, 30, 37 and 42 °C) and pH (5, 6, 7, 8 and 9). The effect of initial Cd^2+^ concentration on the Cd^2+^ processing ability was checked by growing the isolate in different initial concentration of Cd^2+^ ranging from 0.5 to 5 mM. Presence of competing metal ions is known to have effect on the biosorption ability. Their effect was studied by growing the isolate in the presence of 1 mM Cd^2+^ along with 1 mM of one of the competing ions (Cr^6+^, Pb^2+^ and Cu^2+^). A mixture of four metal ions (Cd^2+^, Cr^6+^, Pb^2+^ and Cu^2+^) was also used to observe co-metal ions effect. To study the effect of biomass concentration, bacterial isolate was grown in the presence of different initial biomass concentration ranging from 5 to 50 g/L. A blank solution containing only Cd^2+^ was also run as a control in each experiment. After 6 days of incubation, samples (5 mL) were collected and centrifuged at 3000 rpm for 5 min. Pellets were separated, dried and weighed whereas supernatants were used to estimate Cd^2+^ by atomic absorption spectrophotometer. All the experiments were performed in triplicates.

### Biosorption isotherms, thermodynamics and kinetics

The amount of Cd^2+^ removed by the living and dead biomass of the bacterial isolate at time *t* was calculated by the following equation;

$$q = \frac{{V(C_{i} - C_{f} )}}{m}$$

where *q* is Cd^2+^ uptake (mM of Cd^2+^ removed/g of biomass) at time t, *V* is sample volume taken (mL), *C*_*i*_ is the Cd^2+^ concentration initially added in the medium (mM), *C*_*f*_ is the final Cd^2+^ concentration (mM) remained in the medium after time t and *m* is the weight of bacterial biomass (g).

The adsorption capacity at equilibrium (q_e_) was calculated by using following equation.

$$q_{e} = \frac{V(Ci - Ce)}{m}$$

where Ce is the Cd^2+^ concentration at equilibrium.

The Langmuir and Freundlich isotherm equations were applied to Cd^2+^ uptake data by the bacterial isolate.

Langmuir equation is represented as

$$q_{e} = \frac{{q_{max} bC_{e} }}{{1 + bC_{e} }}$$

The linearized form of the equation is

$$\frac{{C_{e} }}{{q_{e} }} = \frac{{C_{e} }}{{q_{max} }} + \frac{1}{{bq_{max} }}$$

Values of q_max_ and b were calculated by drawing a plot of C_e_/q_e_ vs C_e_.

where q_e_ refers to the amount of metal ions sorbed at equilibrium (mM/g), C_e_ is the metal ions concentration in solution at equilibrium (mM), q_max_ is the maximum sorption capacity of biosorbent (mM/g) and b is Langmuir constant.

A dimensionless constant separation factor (R_L_) was calculated to express the Langmuir isotherm feasibility by using following equation;

$$R_{L} = \frac{1}{{1 + bC_{i} }}$$

where b is Langmuir constant and C_i_ is the initial concentration of metal ion (Cd^2+^).

Freundlich isotherm model reveals the sorption of metal ions on heterogenous surfaces forming multilayers. Freundlich equation is written as

$$q_{e} = k_{f} C_{e}^{{\frac{1}{n}}}$$

Its linearized form is written as

$$\ln \,\,q_{e} = \ln K_{f} + \frac{1}{n} {\ln}C_{e}$$

The values of K_f_ and 1/n can be calculated by drawing plot of ln q_e_ vs ln C_e_.

Standard thermodynamic parameters i.e. Gibbs free energy change (∆G°), enthalpy change (∆H°) and entropy change (∆S°) were calculated by using following equations;

$$\Delta {\text{G}}^\circ = - {\text{RTln}}K_{D}$$

$${\ln}\, K_{D} = \frac{{(\Delta {\text{S}}^\circ ) }}{R} + \frac{{(\Delta {\text{H}}^\circ ) }}{RT}$$

where R is universal gas constant (8.314 J/mol K), T refers to temperature (K) and K_D_ (q_e_/C_e_) is distribution coefficient. The values of ∆S° and ∆H° were calculated by drawing plot of ln K_D_ vs 1/T.

The dynamics of Cd^2+^ adsorption by bacterial cells were evaluated by applying pseudo first order and pseudo second order models. The linearized form of pseudo first order model is

$$\ln \left( {q_{e} - q_{t} } \right) = {\ln}q_{e} - k_{1} t$$

where q_e_ and q_t_ are the adsorption capacity of Cd^2+^ at equilibrium and given time, t is temperature and k_1_ is rate constant. The value of k_1_ was calculated by drawing plot of ln(q_e_−q_t_) vs t.

The linearized form of pseudo second order model is;

$$\frac{t}{{q_{t} }} = \frac{t}{{k_{2} (q_{e}^{2} )}} + \frac{1}{{q_{e} }}$$

k_2_ (second order rate constant) was calculated by plot of t/q_t_ vs t.

### Estimation of glutathione (GSH) and other non-protein thiol contents

Reduced and oxidized GSH and total thiol contents were estimated according to Shamim and Rehman (2015).

### Protein extraction and SDS–PAGE

Bacterial total protein profiling was done according to Laemmli (1970).

### Amplification of smtAB

An internal fragment of 480 bp of known *smtAB* gene was amplified from both plasmid and genomic DNA through PCR using known primers (smtAB-F; 5′-GAT CGACGTTGCAGA GACAG-3′, smtAB-R; 5′-GATCGAGGGCGTTTTGATAA-3′) reported by Naz et al. (2005). The reaction mixture (20 µL) contained 0.2 mM dNTPs, 20 pM each primer, 10 ng DNA, 0.25 U taq polymerase, 50 mM KCl buffer and 1.5 mM MgCl_2_. Amplification was carried out by 35 cycles of denaturation at 94 °C for 1 min, annealing at 56 °C for 1 min and extension at 72 °C for 2 min followed by final extension at 72 °C for 5 min.

### Fourier transform infrared spectroscopy (FTIR)

For FTIR analysis bacterial samples were prepared according to Deokar et al. (2013). Briefly, the bacterium was grown in the presence and absence of 1 mM Cd^2+^ for 24 h at 37 °C. The bacterial cells were harvested by centrifugation at 3000 rpm for 10 min and washed several times with saline solution (0.9 % NaCl, pH 6.5). Then bacterial pellets were freeze-dried overnight and their infrared spectra were recorded on a FTIR spectrometer (Bruker, alpha) in the region 4000–500/cm.

### Scanning electron microscopy (SEM)

To prepare samples for scanning electron microscopy a drop of bacterial suspension (both untreated and treated with 1 mM Cd^2+^) was mounted on aluminum stub and air dried. Bacterial cells were fixed with 2.5 % glutaraldehyde in phosphate buffer saline (pH 7.2), incubated at room temperature for 30 min and washed thrice with Na-phosphate buffer. Samples were dehydrated with graded acetone series (30, 50, 70, 80, 90 and 100 %) for 10 min for each step. For better contrast, samples were coated with gold film by sputter coater (Denton, Desk V HP) operating at 40 mA for 30 s under vacuum and analyzed by scanning electron microscope (Nova NanoSEM 450) equipped with Oxford energy dispersive X-ray (EDX) microanalysis system.

### Statistical analysis

Observations were made and all the experiments run in triplicate. At least three separate flasks were usually maintained for one treatment. Each time three readings were taken, their mean, and standard error of the mean were calculated.

The sequences of the 16S rRNA gene was submitted to Genbank under the accession number of KJ880038.

## Results

### Physiochemical characteristics of wastewater

Some physicochemical parameters of wastewater were measured at the time of sampling. Temperature of different samples ranged between 25 and 40 °C, pH was between 6.2 and 9.0 and Cd^2+^ ranged between 0.0042 ± 0.01 and 0.0139 ± 0.01 mM.

### Screening of Cd^2+^ resistant bacteria

The wastewater samples were spread on LB agar plates supplemented with 1 mM Cd^2+^. The Cd^2+^ concentration was increased gradually and a bacterium which showed highest resistance against Cd^2+^ (13.3 mM) was selected for further studies and dubbed as 43C.

### Identification of bacterial isolate

Bacterial isolate was identified on the basis of morphological, biochemical characteristics and 16S rRNA ribotyping. Morphological and biochemical characteristics of the isolate are given in Table 1. The partial sequence of 16S rRNA gene showed 99 % homology with 16S rRNA sequence of *Salmonella enterica* (Accession number N890524.1) already submitted to NCBI database. Interestingly its identity with *S. enterica* subsp. *enterica* serovar *typhimurium* (ATCC 13311) is nearly 93 %, therefore, it can be regarded as non-pathogenic. The sequence of the 16S rRNA gene was submitted to Genbank under the accession number of KJ880038. The dendrogram on the basis of homology was also created (Additional file 1: Figure S1). The organism has been deposited at First Fungal Culture Bank of Pakistan with FCBP no. 590.

**Table 1:** Morphological and biochemical characteristics of *S. enterica* 43C

Form	Irregular
Surface	Smooth
Color	Off white
Margin	Entire
Elevation	Flat
Opacity	Opaque
Gram staining	Ve−
Catalase	Ve+
Oxidase	Ve−
Citrate	Ve−
Lactose fermentation	Ve−
H_2_S production	Ve−
Nitrate	Ve−
Indole	Ve−
Urease	Ve−
Voges Proskauer (VP)	Ve−

*Ve*− negative, *Ve*+ positive

### Optimum growth conditions

**Fig. 1:**
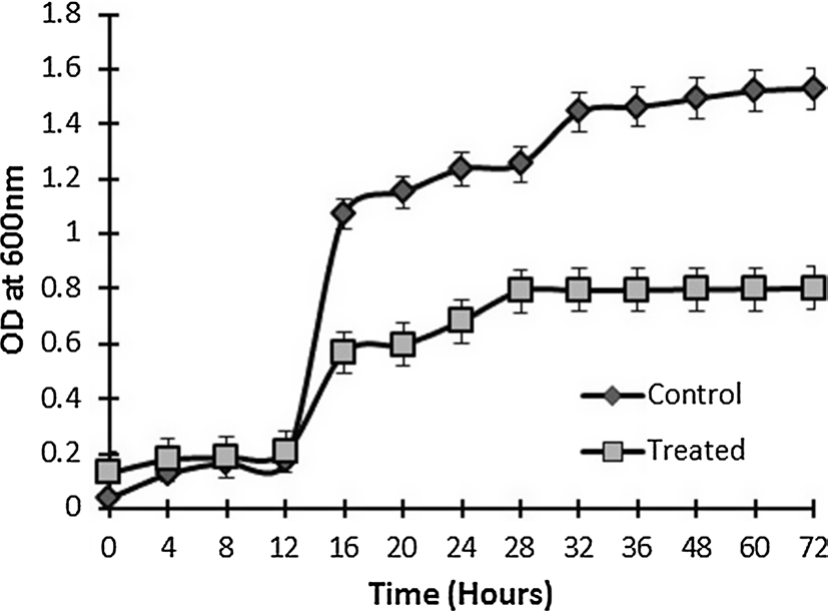
Growth pattern of *S. enterica* 43C in the absence (control) and presence (treated) of 1 mM Cd^2+^

The most suitable temperature for *S. enterica* 43C was found to be 37 °C and it revealed maximum growth at pH 7. The effect of Cd^2+^ on the growth of *S. enterica* 43C was monitored by growing it in the presence (1 mM Cd^2+^) and absence of Cd^2+^. The presence of Cd^2+^ significantly decreased the growth of the bacterium (Fig. 1).

### Cross metal resistance

The bacterium resisted Cd^2+^ up to 13.3 mM. It was also capable to resist other heavy metal ions, viz., Zn^2+^ (5.3 mM), Pb^2+^ (16 mM), Cu^2+^ (2.6 mM), Cr^6+^ (5.3 mM), As^3+^ (8.8 mM) and Hg^2+^ (0.53 mM). The order of resistance regarding metal ions concentration was Pb^2+^>Cd^2+^>As^3+^>Zn^2+^>Cr^6+^>Cu^2+^>Hg^2+^.

### Biosorption of Cd^2+^

**Fig. 2:**
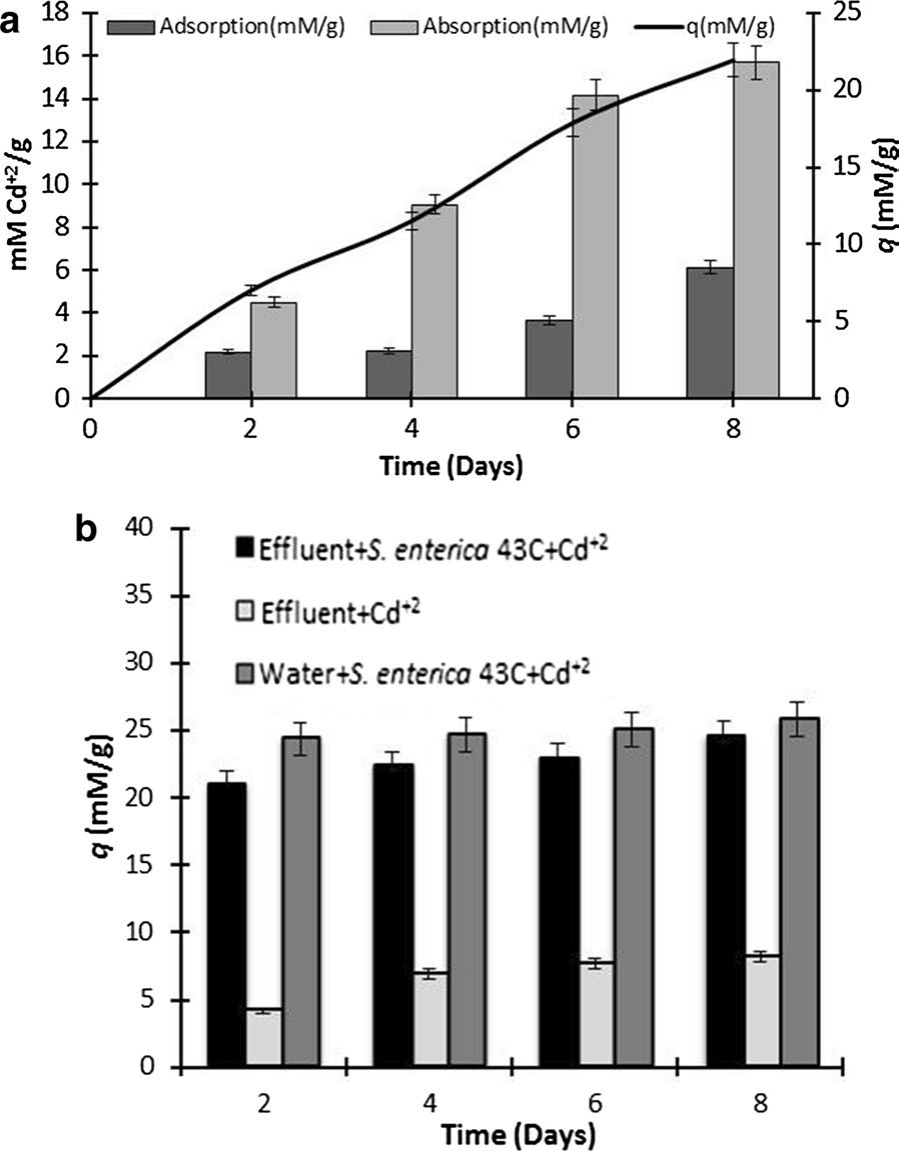
**a** Biosorption capability of *S. enterica* 43C growing in culture medium supplemented with 1 mM Cd^2+^ and **b** large scale biosorption of Cd^2+^ by *S. enterica* 43C

Biosorption capability of *S. enterica* 43C was assessed by growing it in culture medium supplemented with 1 mM Cd^2+^ (Fig. 2a). Cd^2+^ biosorption efficiency (*q*) of *S. enterica* 43C was as 7, 11.5, 18 and 22 mM/g after 2, 4, 6 and 8 days, respectively. The amount of Cd^2+^ found accumulated within bacterial cells after 2, 4, 6 and 8 days was 4.5, 9, 14 and 15.7 mM/g, respectively. While 2.18, 2.2, 3.6 and 6 mM/g of Cd^2+^ was found to be adsorbed on the bacterial surface after 2, 4, 6 and 8 days, respectively (Fig. 2a).

At large scale *S. enterica* 43C was able to remove 41, 43, 45.8 and 49 mM/g 83.4 % Cd^2+^ from industrial effluent and 43.8, 45, 50 and 51.5 mM/g from distilled water after 2, 4, 6 and 8 days, respectively. Cd^2+^ concentration was maintained at 1 mM in both water and industrial effluent. The microorganisms originally present in industrial effluent could remove 4, 9, 12 and 16 mM/g after 2, 4, 6 and 8 days, respectively from original industrial effluent in which no Cd^2+^ was added but originally contained 0.18 mM Cd^2+^ (Fig. 2b).

### Cd^2+^ removal by heat-inactivated bacterial cells

Bacterial cells were inactivated by autoclaving to use as biosorbant material for Cd^2+^ bioremediation. Dead cells of *S. enterica* 43C were observed removing 8.8, 15, 21 and 23.8 mM/g Cd^2+^ from aqueous medium, out of which 4.27, 7.2, 10.84 and 11.06 mM/g Cd^2+^ was detected adsorbed on outer surface whereas 4.91, 7.92, 10.6 and 12.79 mM/g Cd^2+^ found accumulated within dead yeast cells after 2, 4, 6 and 8 days, respectively (Additional file 1: Figure S2).

### Biosorption isotherms, thermodynamics and kinetics

**Fig. 3:**
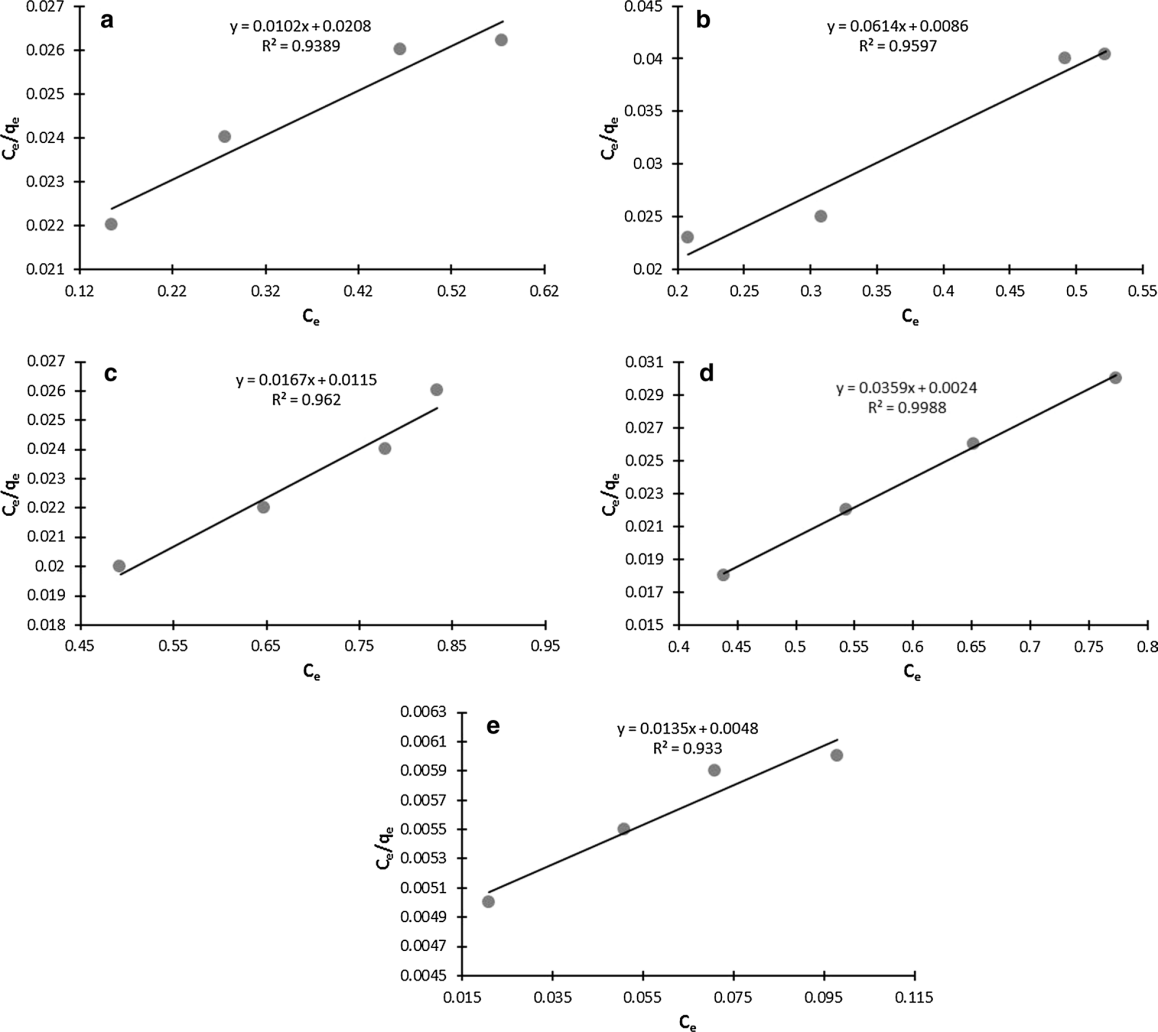
Langmuir isotherm for **a** living cells **b** dead cells **c** effluent plus *S. enterica* 43C plus Cd^2+^ (1 mM) **d** distilled water plus *S. enterica* 43C plus Cd^2+^ (1 mM) **e** only effluent

**Fig. 4:**
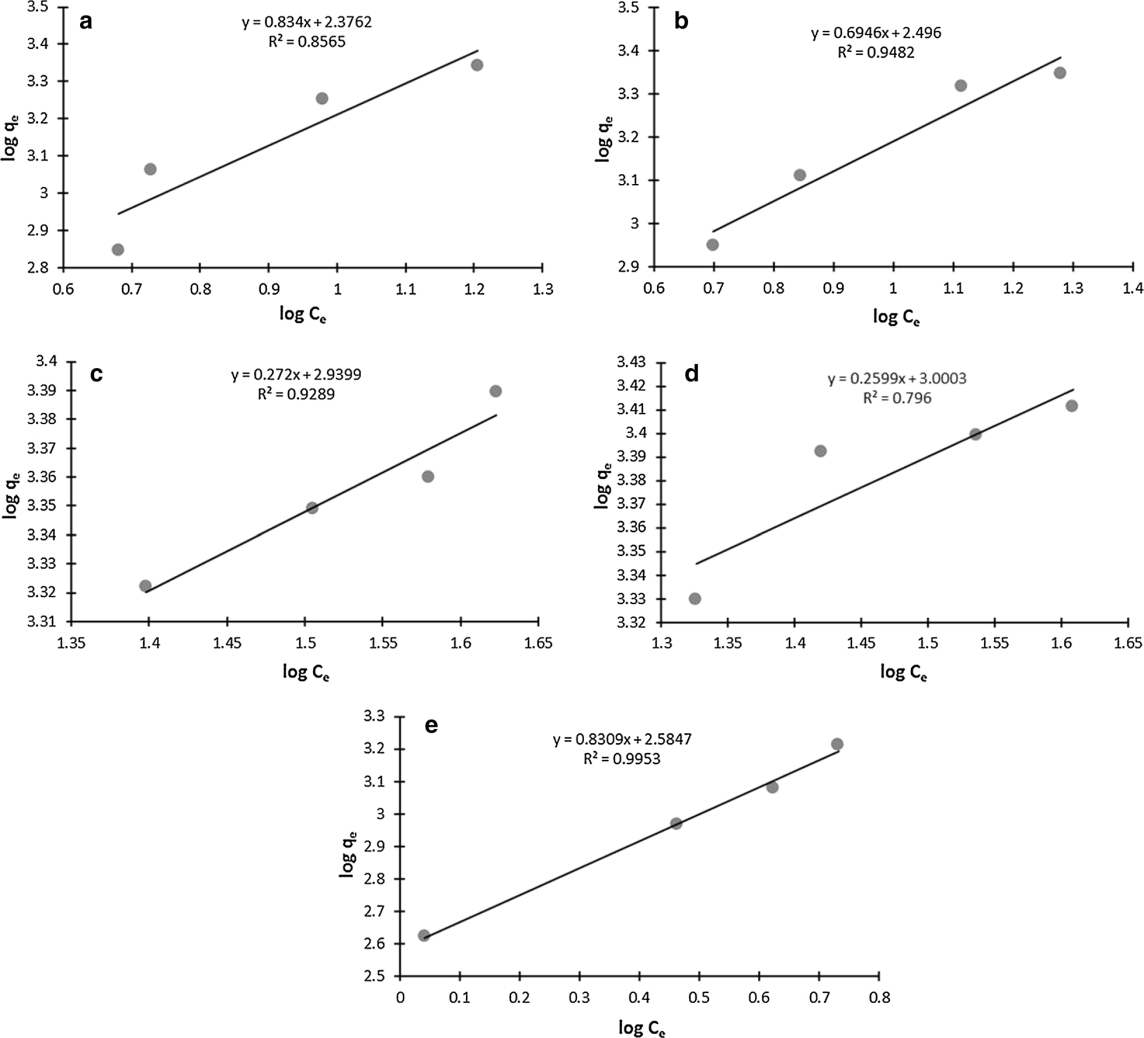
Freundlich isotherm for **a** living cells **b** dead cells **c** effluent plus *S. enterica* 43C plus Cd^2+^ (1 mM) **d** distilled water plus *S. enterica* 43C plus Cd^2+^ (1 mM) **e** only effluent

Straight lines were obtained when we evaluated our data by applying Langmuir and Freundlich isotherm equations (Figs. 3, 4a–e) which suggested that our data is correlated with both the isotherm models. The values of Langmuir constants (q_max_ and b) and Freundlich constant (K_f_ and n) are given in Additional file 1: Table S1. The regression coefficient (R^2^) values for Langmuir isotherm ranged 0.933–0.999 whereas these values were found in the range of 0.79–0.995 for Freundlich isotherm (Additional file 1: Table S1) which is an indication that our data is more correlated with Langmuir isotherm model than Freundlich isotherm model. The values of R_L_ (dimensionless constant separation factor which expresses the Langmuir isotherm feasibility) were calculated to determine the suitability of Langmuir model and found in the range of 0.11–0.66 (Additional file 1: Table S1).

The standard Gibbs free energy change (∆G°) was calculated as −4.96, −5.4, −6.9, −7.5, −7.7 and −8.3 kJ/mol at 20, 25, 30, 33, 37 and 42 °C, respectively. Whereas, standard enthalpy (∆H°) and entropy (∆S°) changes were 48.9 kJ/mol and 216.4 J/mol K, respectively (Additional file 1: Table S2) from plot of ln K_D_ vs 1/T (Additional file 1: Figure S3).

**Fig. 5:**
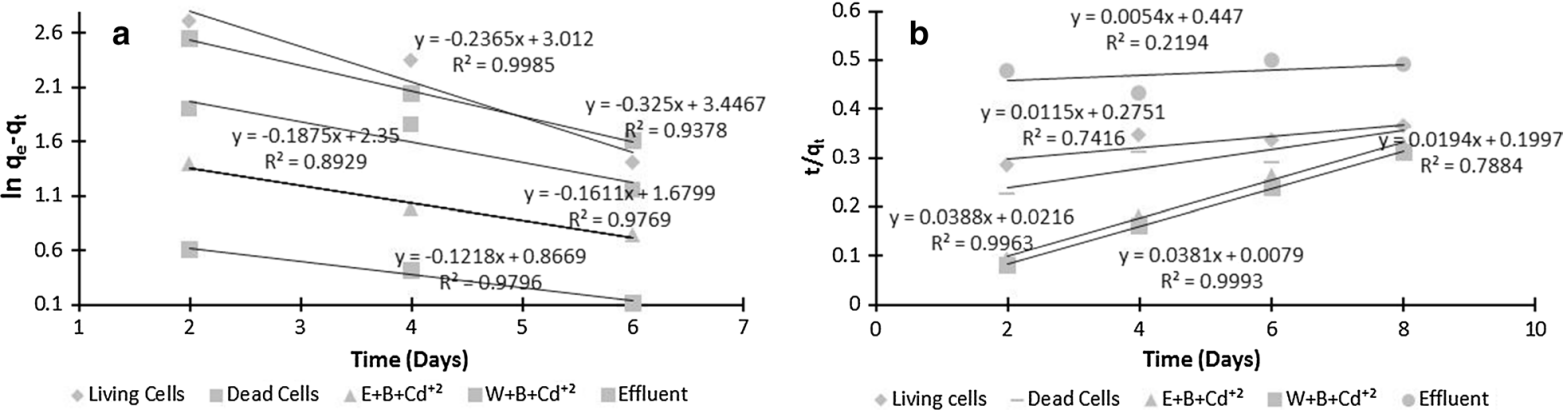
Pseudo first order (**a**) and pseudo second order (**b**) reaction for Cd^2+^ adsorption onto *S. enterica* 43C

Adsorption data was evaluated by applying pseudo first and pseudo second order kinetic models. The values of constants (*K*_1_ and *K*_2_) and coefficient determinant (R^2^) are given in Additional file 1: Table S3. Pseudo first order R^2^ values are higher for lab scale experiments (0.9) than pseudo second order R^2^ values. Figure 5a, b showing plots of q_t_/t vs time(t). Furthermore, experimental *q* values (*q*_*exp*_) 21 and 9 mM/g were found in good agreement with calculated *q* values (*q*_*cal*_) 31 and 10 mM/g for living and dead cells, respectively by using pseudo first order equation. Whereas, at large scale experiments pseudo second order R^2^ values were higher (0.99) than pseudo first order R^2^ values (0.97). Moreover, q_exp_ values and q_cal_ values (calculated by using pseudo second order equation) were exactly similar for industrial effluent and distilled water. In both of them bacterial biomass and Cd^2+^ (1 mM) were added. In general, these results suggest that lab scale experiments follow pseudo first order whereas large scale experiments follow pseudo second order model. But the original effluent in which no bacterial biomass and Cd^2+^ were added was found following pseudo first order kinetic model just like small scale experiments.

### Effect of temperature, pH, initial Cd^2+^ concentration, biomass dosage and co-metal ions on Cd^2+^ processing ability of bacterial isolate

**Fig. 6:**
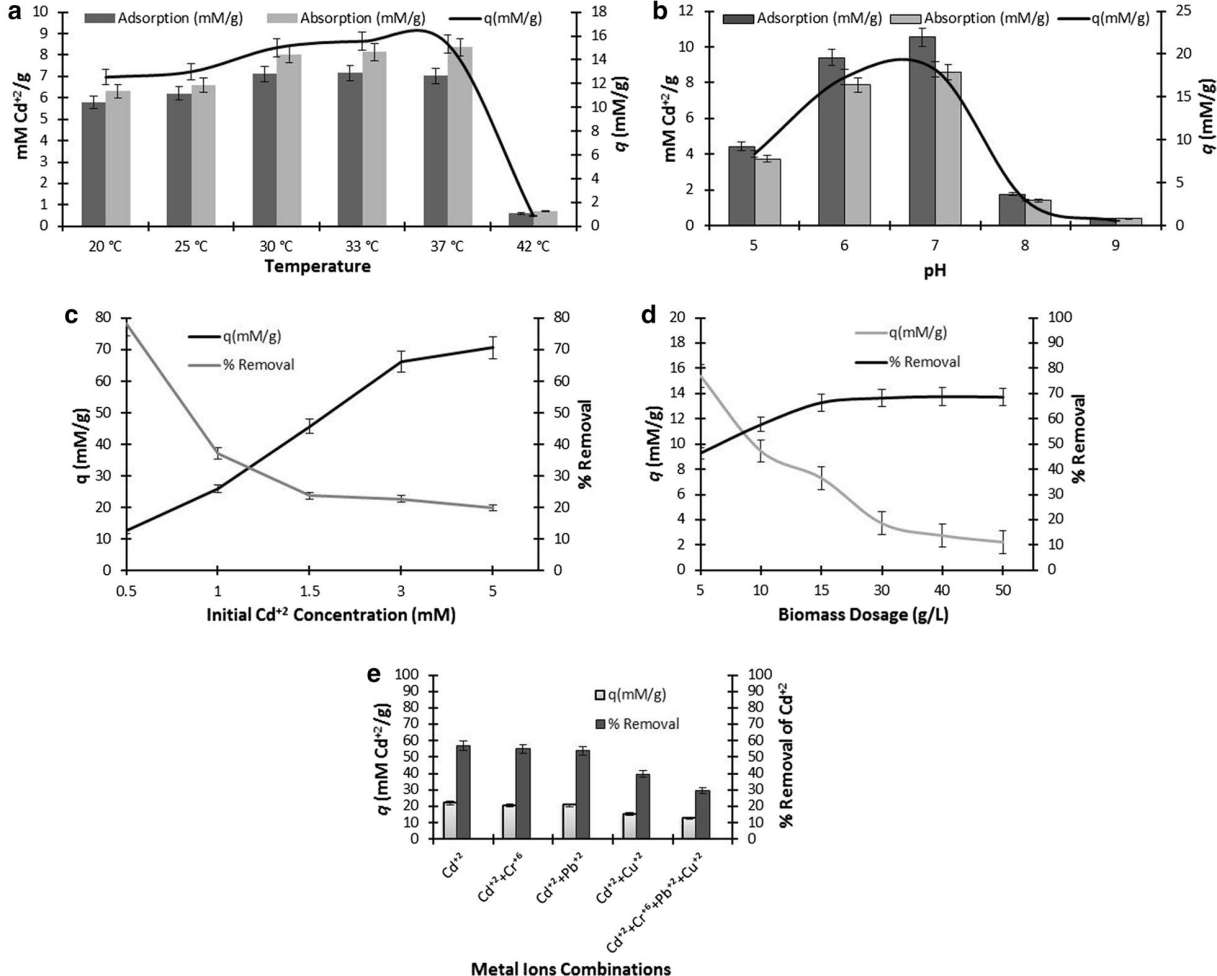
Effect of temperature (**a**), pH (**b**), initial Cd^2+^ concentration (**c**), biomass dosage (**d**) and co-metal ions (**e**) on Cd^2+^ processing ability of *S. enterica* 43C

Several parameters have been proved to have influence on the Cd^2+^ processing ability of *S. enterica* 43C. It showed optimum biosorption capability at moderate temperature (30 to 37 °C) where 15 mM/g removal of Cd^2+^ was observed. This removal was reduced to 0.89 mM/g when temperature was increased up to 42 °C. Similarly at low temperature (25 °C), it could remove only 13 mM/g Cd^2+^ from the medium (Fig. 6a). The biosorption ability of *S. enterica* 43C was also found changing with varying pH. Neutral (pH 7) or nearly neutral pH (pH 6) was found most suitable for *S. enterica* 43C and it removed 18 and 17.3 mM/g Cd^2+^, respectively. Basic (pH 8) and strongly basic pH (pH 9) decreased Cd^2+^ removal to 2.9 and 0.53 mM/g, respectively. At slightly acidic pH (pH 5), it exhibited 8.3 mM/g removal (Fig. 6b). The sorption of Cd^2+^ by *S. enterica* 43C was studied by varying initial Cd^2+^ concentration ranging from 0.5 to 5 mM. A direct relationship was observed between initial Cd^2+^ concentration and Cd^2+^ processing ability of *S. enterica* 43C 12.3, 25.8, 45, 66 and 71 mM/g sorption of Cd^2+^ by *S. enterica* 43C was recorded at 0.5, 1, 1.5, 3 and 5 mM of initial Cd^2+^ concentration, respectively (Fig. 6c). Figure 6d is indicating the effect of bacterial biomass dosage on the potential of *S. enterica* 43C to biosorp Cd^2+^. An increase in Cd^2+^ removal was evaluated with the increase in biomass concentration but up to a certain limit. *Salmonella enterica* 43C showed 46, 58, 66, 68 %, removal at 5, 10, 15 and 30 mg/mL of initial biomass concentrations, respectively. But biosorption efficiency (*q*) was decreased from 15 to 2 mM/g. Further increase (40 and 50 mg/mL) in biomass concentration did not cause any significant change in Cd^2+^ removal efficiency of *S. enterica* 43C. When *S. enterica* 43C was allowed to grow in the presence of Cd^2+^ in combinations with different heavy metal ions as competing ions, a change in bioremediation potential of *S. enterica* 43C was noticed. *S. enterica* 43C could remove 22.8, 26.6 and 12.2 mM/g Cd^2+^ when Cr^6+^, Pb^2+^ and Cu^2+^ were used as competing ions, respectively. This removal capacity was decreased to 11.7 mM/g when all these heavy metal ions i.e. Cd^2+^, Cr^6+^, Pb^2+^ and Cu^2+^ were added simultaneously. It is kept in mind that *S. enterica* 43C showed 21.8 mM/g removal of Cd^2+^ in the absence of any competing ion (Fig. 6e).

### Measurement of glutathione and non-protein thiols

The levels of GSH and GSSG were altered in the presence of Cd^2+^ (1 mM). There was 53.96 and 154 % increase in GSH level and GSH/GSSG ratio, respectively. Whereas 87 % increase in non-protein thiols level was recorded in bacterial cells in the presence of Cd^2+^ (Additional file 1: Table S4).

### SDS–PAGE analysis

**Fig. 7:**
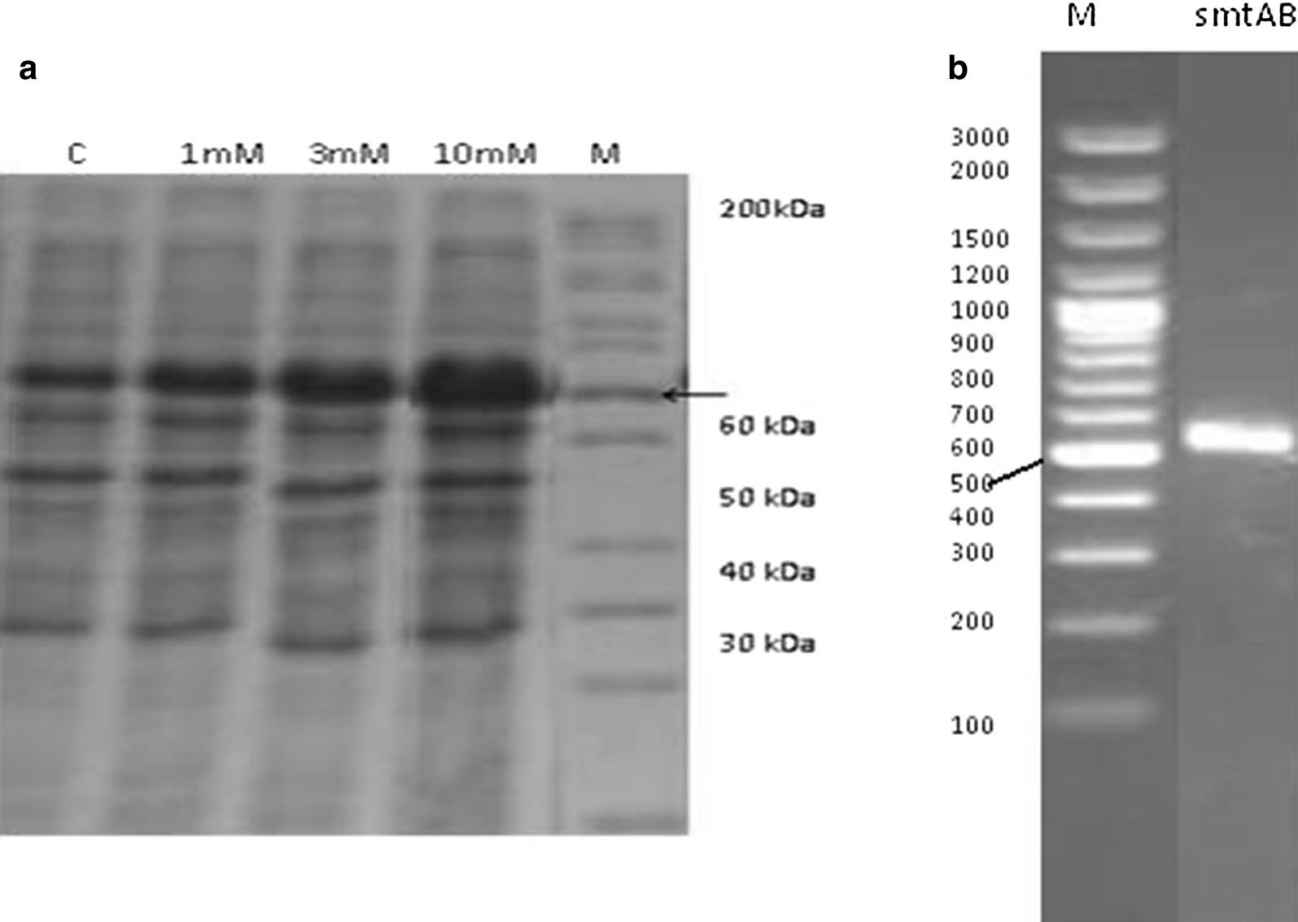
**a** Total protein profile of *S. enterica* 43C in the absence and presence of 1, 5 and 10 mM Cd^2+^. *M* protein marker; *C* control (without Cd^2+^) and **b** amplification of *smtAB* gene through PCR. M represents DNA ladder

Total protein profiles of *S. enterica* 43C in the absence and presence of Cd^2+^ were compared by polyacrylamide gel electrophoresis. This comparison clearly indicated the induction of a protein due to Cd^2+^ stress. A band of about 40 kDa was observed intensifying with the increasing concentration of Cd^2+^ (Fig. 7a).

### Amplification of smtAB

PCR was carried out to amplify internal fragment of *smtAB* gene from both plasmid and genomic DNA of *S. enterica* 43C by using known primers. The primer set showed amplification of 480 bp product. But no amplification was detected when plasmid DNA was used as a template (Fig. 7b).

### SEM, EDX and FTIR

**Fig. 8:**
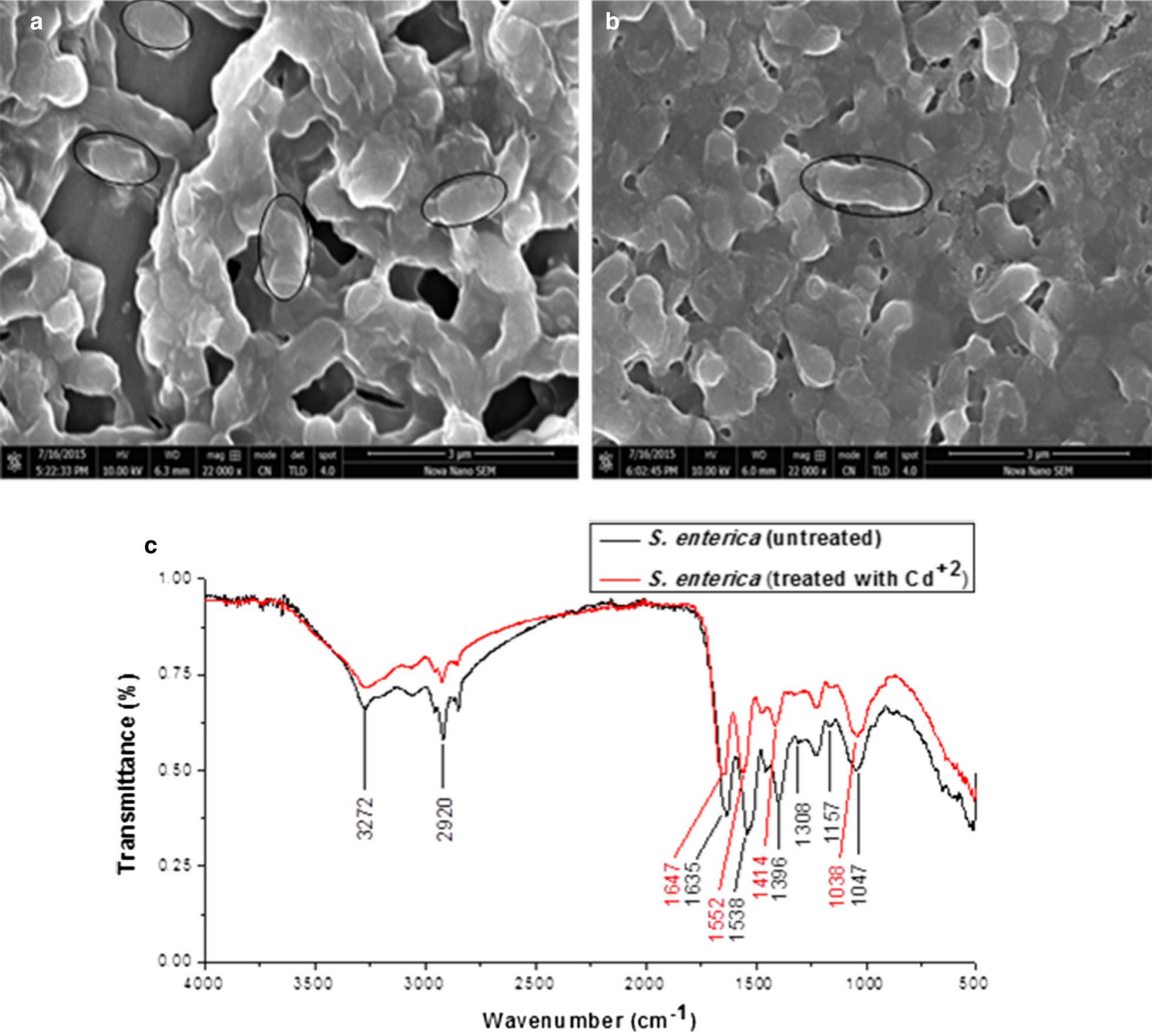
Images of scanning electron microscopy of *S. enterica* 43C in the absence (**a**) and presence (**b**) of Cd^2+^ (1 mM) and **c** FTIR of *S. enterica* 43C in the absence and presence of Cd^2+^

The size of *S. enterica* 43C was observed to increase many folds, when treated with Cd^2+^. This indicated the accumulation of Cd^2+^ and it was confirmed from the difference in size, when SEM images of treated *S. enterica* 43C were compared with untreated bacterium (Fig. 8a). This biosorption of Cd^2+^ was verified by EDX (point and ID scan) analysis, while doing their SEM (Additional file 1: Figure S4). The FTIR analysis confirmed the presence of carboxyl, amino and phosphate moieties in bacterium and this analysis confirmed our assumption regarding binding of Cd^2+^ with bacterium. It is clear that the peaks attributed to amide linkage, appearing at 1635 and 1538/cm are shifted to 1647 and 1552/cm respectively, in the presence of Cd^2+^ (Fig. 8b).

## Discussion

In the present investigation we have demonstrated the potential use of *S. enterica* 43C to remove Cd^2+^ from aqueous medium at lab scale. *S. enterica* 43C could remove maximally 22 mM/g Cd^2+^ from the aqueous medium and 2.18, 2.2, 3.6 and 6 mM/g Cd^2+^ were found to be adsorbed on bacterial cell surface after 2, 4, 6 and 8 days, respectively. Whereas *Pseudomonas stutzeri* and *Enterobacter* sp. have been reported to remove 43.5 and 46.2 mg/g Cd^2+^ by adsorption (Lu et al. 2006). At large scale, *S*. *enterica* 43C maximally removed 24 mM/g (83 %) and 25 mM/g (77 %) after 8 days from effluent and water supplemented with 1 mM Cd^2+^. Effluent naturally contains some amount of heavy metal ions as well as number of microorganisms including bacteria, fungi, and protozoans. Experiment was also performed to check the efficacy of these naturally existing microbes to remove Cd^2+^. Results showed that these organisms could maximally remove only 27 % Cd^2+^. But our bacterium, *S. enterica* 43C removed 83 % Cd^2+^ suggesting its preferential use over other microbes for Cd^2+^ bioremediation.

There are several adsorption models but Langmuir and Freundlich models are commonly used (Bayramoğlu et al. 2006; Ansari and Malik 2007; Kang et al. 2007) which are based on monolayer and multilayer adsorption, respectively. In Langmuir model we make some assumptions that biosorbent surface is uniform and metal ions do not have mutual interaction and adsorb on the biosorbent surface in single layer. Additional file 1: Table S1 is showing the values of different parameters for Langmuir and Freundlich isotherm models, higher values of regression coefficient for Langmuir isotherm model indicated that our data was more consistent with Langmuir model than Freundlich model. Langmuir model is considered unfavorable for a given data if R_L_ >1, while it is favorable when 0 < R_L_ < 1 (Hall et al. 1966). In our experiments, R_L_ values were found less than 1 and greater than 0 (0.11–0.66), indicating the favorability of Langmuir isotherm model. High biosorption capacity of *S. enterica* 43C (either living or dead form) for Cd^2+^ as compare to other biosorbent reported in literature (Say et al. 2001; Martins et al. 2004; Sari et al. 2008; Chatterjee et al. 2010) to bioremediate Cd^2+^ makes it highly potential and favorable resource to remove environmental Cd^2+^.

SEM revealed the increase in the size of bacterial cells in the presence of Cd^2+^ which clearly indicated the biosorption of Cd^2+^. It was further confirmed by EDX and FTIR. The characteristic peaks of carboxyl, amino and phosphate groups appearing in FTIR analysis confirmed the presence of these moieties in bacterium and the shift in these peaks was used to address the binding of Cd^2+^ with bacterium. In short, the peaks at 1635 and 1538/cm recognized the presence of amide linkage, and the shift in these peaks to 1647 and 1552/cm respectively was due to chemical interaction of Cd^2+^ with amide group of bacterium. It is presumed that cadmium mainly adsorbed on bacterium by interacting with nitrogen atoms of amide groups. The peaks appearing in range of 1000–1320/cm represented the presence of carbon and phosphorous containing oxygen atoms, and suppression in intensity of these peaks indicated their interaction with Cd^2+^ (Parikh and Chorover 2006). The peak at 1047 and 1157/cm recognized the presence of carbonyl group and the shift in these peaks and transmittance level was due to chemical interaction of Cd^2+^ with carbonyl moieties. Addition to this, the percentage transmittance of peaks in FTIR spectra of bacterium treated with cadmium, was considerably less significant than those of control bacterium. This showed that stretching of bonds, occurred to lesser degree (due to presence of Cd^2+^), resulted to reduce their percentage transmittance as reported by Chakravarty and Banerjee (2012a).

Biosorption is really a complex process and dependent on lot of factors such as functional groups, temperature, pH, metal ions concentration, weight of biomass and presence of competing metal ions (Lesmana et al. 2009). Among all the factors, pH and temperature have been reported as the most critical parameters in the biosorption of Cd^2+^ (Chakravarty and Banerjee 2012). Present investigation clearly revealed the effect of acidic, basic and neutral pH on the Cd^2+^ processing ability of the bacterium. Strong acidic and strong basic pH significantly decreased the biosorption of *S. enterica* 43C. Maximum biosorption of Cd^2+^ (57.14 %) has been observed at neutral pH. These results are parallel to the findings of Chakravarty and Banerjee (2012). Change in temperature also greatly influences the adsorption and overall biosorption of Cd^2+^. Increase in temperature not only enhances the kinetic energy of reacting molecules but also vibrational energy of constituent elements of bacterial envelop. At high temperature (above 37 °C) the vibrational energy becomes too violent resulting in the breakage of bonds between Cd^2+^ and functional groups on bacterial surface thus decreasing adsorption and intracellular accumulation of Cd^2+^ (Özdemir et al. 2009). Whereas at low temperature chances for reacting molecules to collide with each others are low, thus Cd^2+^ processing ability of the bacterium is declined. *S. enterica* 43C showed maximum Cd^2+^ removal (15 mM/g) at 37 °C but when the temperature was increased up to 42 °C the Cd^2+^ removal capability of *S. enterica* 43C was significantly reduced.

Initial biomass dosage showed significant effect on Cd^2+^ biosorption ability of *S. enterica* 43C. Cd^2+^ biosorption found increasing with the increasing biomass concentration. Highest Cd^2+^ biosorption (68 %) was observed when biomass dosage was increased up to 30 g/L. On contrary, biosorption efficiency (*q*) decreased up to 3.7 mM/g. Further increase in biomass concentration did not cause any significant effect on percentage removal and biosorption efficiency (*q*) of *S. enterica* 43C. Greater concentration of biomass provides greater surface area and binding sites for Cd^2+^ which increases the binding of Cd^2+^ with the biomass and hence increasing the percentage removal of Cd^2+^. However, increase in biomass concentration decreases the ratio of biosorbent to metal ions (as metal ion concentration is fixed i.e. 1 mM) thus a decline in biosorption efficiency (*q*) was observed (Al-Garni 2007).

Industrial wastewater contains multiple metal ions which may influence the removal of Cd^2+^. Present study clearly depicted the effect of other heavy metal ions on Cd^2+^ biosorption in binary and quaternary systems. In multimetal system the binding of metal ions to biomaterial depends on ionic properties, among them electronegativity and ionic radius are the most critical (Naja et al. 2010). Ion with larger radius has greater ability to adsorb and high electronegativity also helps the ion to bind strongly to the surface functional groups (Sulaymon et al. 2011). In the present study, Cd^2+^ biosorption was significantly reduced from 22 to 15 mM/g in the presence of Cu^2+^. Whereas Pb^2+^ and Cr^6+^ showed very slight decrease in Cd^2+^ biosorption in binary system. Electronegativity of Cu^2+^ is 1.9 while Cd^2+^ has 1.7 (Additional file 1: Table S2). This high electronegativity of Cu^2+^ makes it more preferred metal ion over Cd^2+^ to get adsorbed on the surface. Zhou et al. (2009) also reported this preference of Cu^2+^ over Cd^2+^.

*Salmonella enterica* 43C showed tolerance against Cd^2+^ (13.3 mM) as well as against Zn^2+^ (5.3 mM), Pb^2+^ (16 mM), Cu^2+^ (2.6 mM), Cr^6+^ (5.3 mM), As^3+^ (8.8 mM) and Hg^2+^(0.53 mM). The order of resistance regarding metal ions concentration was Pb^2+^>Cd^2+^>As^3+^>Zn^2+^>Cr^6+^>Cu^2+^>Hg^2+^. *Salmonellaenterica* 43C was found to be more tolerant against Pb^2+^ as compared to the Cd^2+^. This is due to the fact that Pb^2+^ is more ubiquitous heavy metal. On the other hand Cd^2+^ is highly toxic and its no physiological role has been reported so far (Martelli et al. 2006; Vinodini et al. 2015). *Salmonella enterica* 43C is notorious for its pathogenic behavior. Anyhow, pathogenicity cannot be considered as a limiting factor to use microorganisms for bioremediation purposes. Number of pathogenic bacteria including *Enterobacter* sp., *P. aeruginosa*, *Shewanella* and *Alcaligenes faecalis* have been used for bioremediation purposes (Lu et al. 2006; Srivastava and Majumder 2008; Kang et al. 2015).

**Fig. 9:**
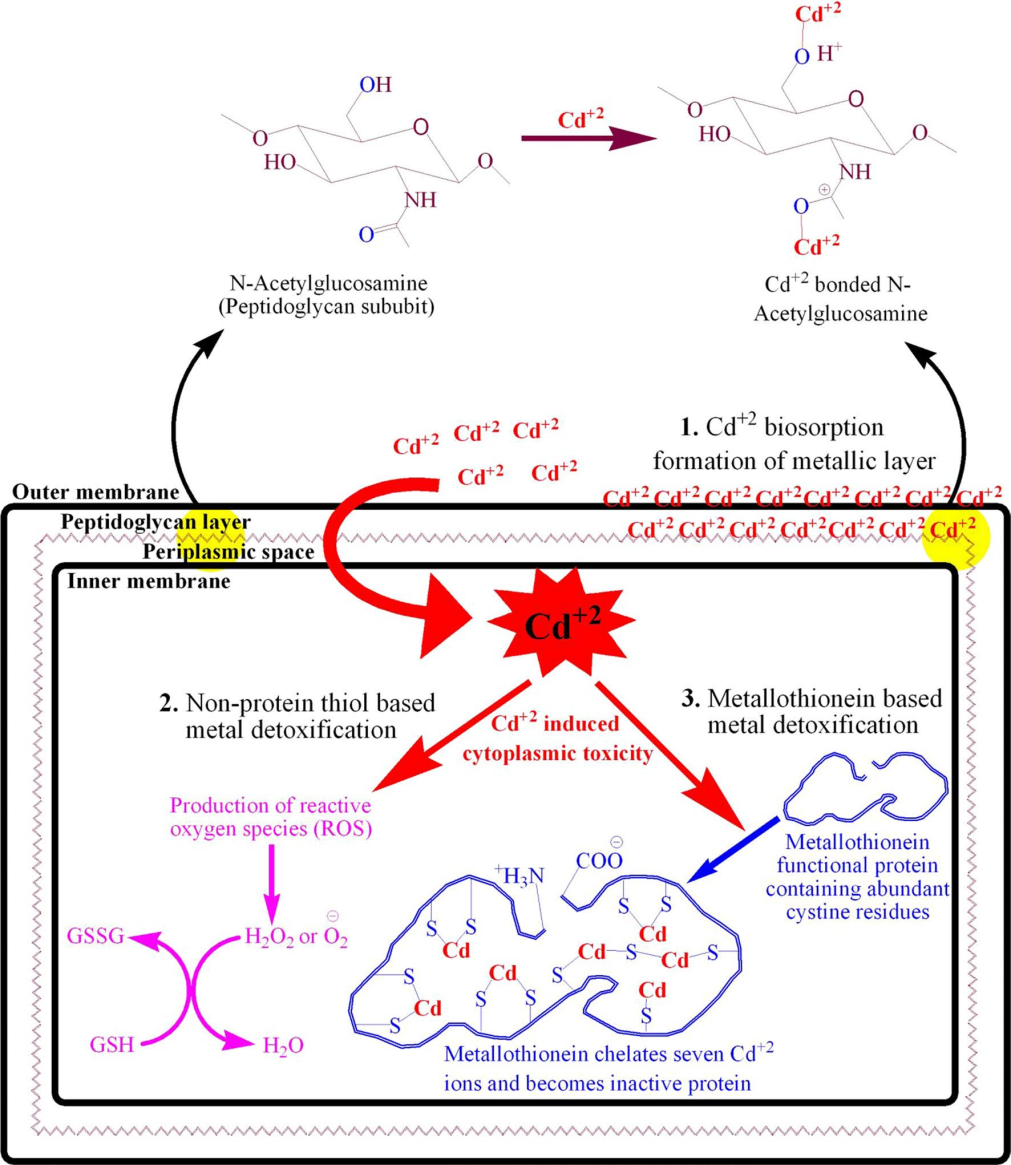
Proposed Cd^2+^ biosorption and resistance mechanism in gram negative bacterium, *S. enterica* 43C. (*1*) Peptidoglycan is the primary site on bacterial surface that binds Cd^2+^ in monolayer. (*2*) Cd^2+^ enter the cells via metal ions transport channel proteins and are known to cause oxidative stress which is combated by GSH dependent antioxidant system. (*3*) Metallothioneins, induced by Cd^2+^ stress, are primarily involved in Cd^2+^ sequestration through their thiols groups thus mitigating Cd^2+^ interference with cellular metabolism

Bacteria have developed different resistance mechanisms to cope with the negative effects of these metal ions (Sinha and Mukherjee 2008; Hassan et al. 2009; Khan et al. 2015) including alteration in the levels of GSH and GSSG and induction of metal binding proteins i.e. metallothioneins (Fig. 9). GSH, as a strong reducing agent, reduces the H_2_O_2_ to H_2_O and gets itself oxidized to GSSG (Penninckx 2002; Galano and Alvarez-Idaboy 2011). GSH/GSSG is the most important parameter to study oxidative stress. Present study clearly revealed the alteration of GSH level and GSH/GSSG. An increase of 53.96 and 154 % in GSH level and GSH/GSSG ratio was determined in the presence of Cd^2+^, respectively. An increase of 87 % in non-protein thiols level was also determined.

MTs are low molecular weight (6–7 kDa), thiol-containing, cysteine rich (20–30 %), metal binding proteins, induced by cadmium and other heavy metal ions and are involved in the sequestration of metal ions (Klaassen et al. 2009; Chaturvedi et al. 2012). MTs are widely associated with eukaryotes (Palmiter 1998) and among prokaryotes, these have been reported in *P. putida* and *Synechococcus* spp. (Higham et al. 1984; Huckle et al. 1993). The amplification of *smtAB* gene from the chromosomal DNA of *S. enterica* 43C revealed the presence of genetic determinants of bacterial metallothioneins. These metallothioneins encoding genes have been found involve in conferring resistance against heavy metal ions including Cd^2+^ (Turner et al. 1995).

In the present study, *S. enterica* 43C showed high resistance against heavy metal ions in order of Pb^2+^>Cd^2+^>As^3+^>Zn^2+^>Cr^6+^>Cu^2+^>Hg^2+^. The bacterium could remove nearly 57 % Cd^2+^ from the medium over a period of 8 days. Kinetic and thermodynamic studies depicted the Cd^2+^ biosorption as spontaneous, feasible and endothermic in nature. The bacterium followed pseudo first order kinetics, making it a good biosorbent for heavy metal ions. The Cd^2+^ processing was significantly influenced by temperature, pH, initial Cd^2+^ concentration, biomass dosage and co-metal ions. FTIR analysis revealed the active participation of amide and carbonyl moieties in Cd^2+^ adsorption which was confirmed by EDX analysis. Electron micrographs beckoned further surface adsorption and increased bacterial size due to intracellular Cd^2+^ accumulation. An increase in GSH and other non-protein thiols levels played a significant role in thriving oxidative stress generated by metal cations. MTs presence clearly indicated the role of such proteins in bacterial metal resistance mechanism. *S. enterica* 43C, a non-pathogenic bacterium having strong cytoplasmic redox balance system to overcome metal induced cytotoxicity. Moreover, its pseudo first order kinetic trends make it an effective biosorbent to remove toxic heavy metals from the aqueous environment. In future, after exploring its molecular biology it can become an attractive environmental tool for green chemistry.

## Additional file

10.1186/s13568-016-0225-9 Tables and figures.

## Abbreviations

LB: Luria–Bertani
PCR: polymerase chain reaction
FTIR: fourier transform infrared
SEM: scanning electron microscopy
EDX: energy dispersive X-ray
GSH: glutathione
MTs: metallothioneins
