# Supplementary material for: Cadmium resistance and uptake by bacterium, Salmonella enterica 43C, isolated from industrial effluent
Source: AMB Express. 2016 Aug 4;6:54. doi: 10.1186/s13568-016-0225-9 (PMC4974214; doi:10.1186/s13568-016-0225-9)
Supplement: Supplementary file 1 — 10.1186/s13568-016-0225-9 Tables and figures. [file 13568_2016_225_MOESM1_ESM.doc]

**Journal: AMB Express**

**Supplementary materials (Tables and figures)**

**Title: Cadmium resistance and uptake by bacterium,** *Salmonella enterica* 43C, **isolated from industrial effluent**

## **Authors:** Zaman Khan,^a^ Abdul Rehman,^a^* Syed Z. Hussain,^b^ Muhammad A. Nisar^c^ , Sumble Zulfiqar^d^, Abdul R. Shakoori^d^

## ^1^Department of Microbiology and Molecular Genetics, University of the Punjab, New Campus, Lahore 54590, Pakistan

^2^Department of Chemistry, SBA School of Science and Engineering (SBASSE), Lahore University of Management Sciences (LUMS), DHA, Lahore Cantt-54792, Pakistan

^3^Department of Microbiology, Govt. College University Faislabad, Pakistan

^4^School of Biological Sciences (SZ,ARS), University of the Punjab, New Campus, Lahore54590, Pakistan

## ***Correspondence author**

## **Dr. AbdulRehman**

Assistant Professor

Department of Microbiology & Molecular Genetics

University of the Punjab, New Campus,

Lahore 54590, Pakistan

Tel: 92-42-9231249

Email: rehman_mmg@yahoo.com

**Table S1**: Values of different parameters of Langmuir and Freundlich isotherm models for Cd^+2^ adsorption.

| **Experiments** | **Langmuir** | | | | **Freundlich** | | |
| --- | --- | --- | --- | --- | --- | --- | --- |
|  | **q_max_** | **b** | **R^2^** | **R_L_** | **n** | **K_f_** | **R^2^** |
| **Living Cells** | 98 | 0.5 | 0.9891 | 0.66 | 1.198 | 10.76 | 0.8565 |
| **Dead Cells** | 16.2 | 7.69 | 0.9410 | 0.11 | 1.439 | 12.13 | 0.9482 |
| **Effluent+Bacteria+Cd^+2^** | 59.8 | 1.47 | 0.9989 | 0.40 | 3.676 | 18.91 | 0.9289 |
| **Water+Bacteria+Cd^+2^** | 27.8 | 1.5 | 0.999 | 0.40 | 3.847 | 20.08 | 0.7960 |
| **Effluent** | 74.07 | 2.81 | 0.8565 | 0.26 | 1.203 | 13.25 | 0.9953 |

**Table S2: Thermodynamic parameters for Cd^+2^ adsorption onto *Salmonella enterica*** 43C.

| ***S. enterica* 43C** | **∆H° (kJ/mol)** | **∆S° (J/mol/K)** | **∆G° (kJ/mol)** | | | | | |
| --- | --- | --- | --- | --- | --- | --- | --- | --- |
|  |  |  | **20 °C (293.15 K)** | **25 °C (298.15 K)** | **30 °C (303.15 K)** | **33 °C (306.15 K)** | **37 °C (310.15 K)** | **42 °C (315.15 K)** |
|  | 48.9 | 216 | -4.95 | -5.47 | -6.92 | -7.54 | -7.74 | -8.93 |

**Table S3: Comparison of experimental and calculated adsorption rate constants and correlation coefficients of pseudo first order and pseudo second order equations.**

| **Experiments** | **q_e_,_exp._(mM/g)** | **Pseudo First Order rate constants** | | | **Pseudo Second Order rate constants** | | |
| --- | --- | --- | --- | --- | --- | --- | --- |
|  |  | ***K*_1_** | **q_e_,_cal._**  **(mM/g)** | **R^2^** | ***K*_2_** | **q_e_,_cal._**  **(mM/g)** | **R^2^** |
| **Living cells** | 21.94 | 0.325 | 31.37 | 0.9378 | 4.8 | 86.95 | 0.7416 |
| **Dead Cells** | 9 | 0.1875 | 10.4 | 0.8929 | 0.0018 | 51.54 | 0.7884 |
| **E+B+Cd^+2^** | 25 | 0.1611 | 5.3 | 0.9760 | 0.06 | 25.77 | 0.9963 |
| **W+B+Cd^+2^** | 26 | 0.1218 | 2.379 | 0.9769 | 0.0014 | 26.246 | 0.9993 |
| **Effluent** | 17 | 0.2365 | 20.32 | 0.9985 | 6.5 | 185.185 | 0.2194 |

**Table S4**: Levels of reduced (GSH) and oxidized glutathione (GSSG), total glutathione, reduced and oxidized glutathione ratio, and non-protein thiols in *S. enterica* exposed to 1mM Cd^+2^.

| Cd^+2^ Concentration (mM) | GSH  (mM g^-1^ FW) | GSSG  (mM g^-1^ FW) | GSH+GSSG  (mM g^-1^ FW) | GSH/GSSG | % increase in GSH/GSSG | Non-protein thiols | % increase in non-protein thiols |
| --- | --- | --- | --- | --- | --- | --- | --- |
| 0.0 | 20.2±0.15^a^ | 3.8±0.07 | 24±0.04 | 5.31±0.06 | 8.19:5.31*100 =154.23% | 3.1±0.7 | 2.7:3.1*100= 87% |
| 1.0 | 31.10±0.14 | 2.30±0.21 | 33.4±0.7 | 13.50±0.12 |  | 5.8±0.9 |  |

^a^±SE (n=3)

Figure S1: Neighbor-joining methods based tree of selected *Salmonella enterica* strains.


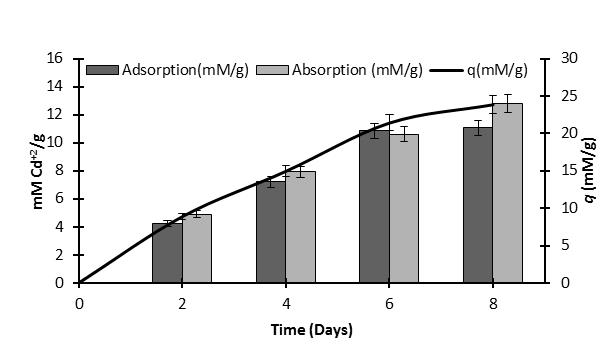


Figure S2: Biosorption of Cd^+2^ by using heat inactivated cells of *S. enterica* 43C.


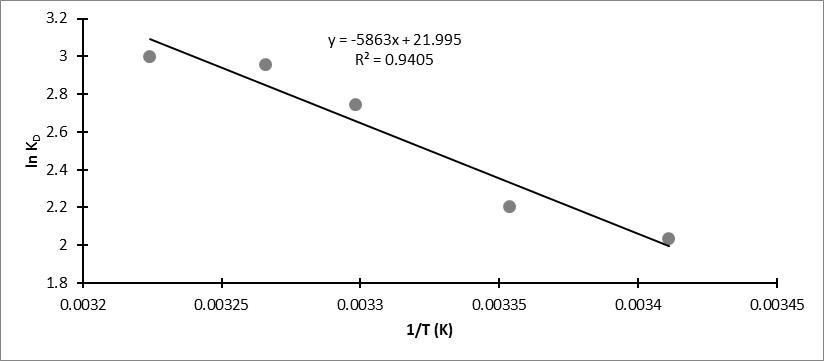


Figure S3: The plot from thermodynamic parameters for Cd^+2^ adsorption onto *S. enterica* 43C were calculated.


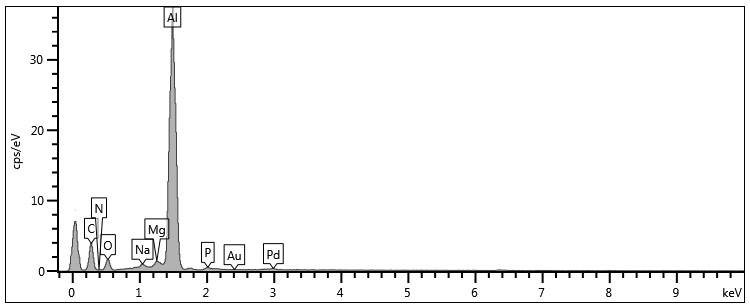


**(a)**

**
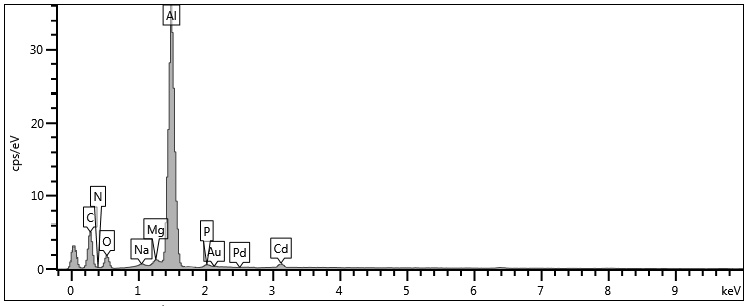
**

**(b)**

Figure S4: Energy dispersive X-ray spectroscopy through SEM of *S. enterica* 43C (a) control and (b) Cd^+2^-treated.
